# Towards a conceptual framework demonstrating the effectiveness of audiovisual patient descriptions (patient video cases): a review of the current literature

**DOI:** 10.1186/1472-6920-12-125

**Published:** 2012-12-21

**Authors:** Damian Roland, Tim Coats, David Matheson

**Affiliations:** 1 Emergency Medicine Academic Group, Emergency Department secretaries c/o Elizabeth Cadman-Moore, Leicester Royal Infirmary, Leicester, LE1 5WW, UK; 2 Room B94C Medical School, Queens Medical Centre, Nottingham, NG7 2UH, UK

**Keywords:** Patient video clips, Methodology, Evaluation, Educational intervention

## Abstract

**Background:**

Technological advances have enabled the widespread use of video cases via web-streaming and online download as an educational medium. The use of real subjects to demonstrate acute pathology should aid the education of health care professionals. However, the methodology by which this effect may be tested is not clear.

**Methods:**

We undertook a literature review of major databases, found relevant articles relevant to using patient video cases as educational interventions, extracted the methodologies used and assessed these methods for internal and construct validity.

**Results:**

A review of 2532 abstracts revealed 23 studies meeting the inclusion criteria and a final review of 18 of relevance. Medical students were the most commonly studied group (10 articles) with a spread of learner satisfaction, knowledge and behaviour tested. Only two of the studies fulfilled defined criteria on achieving internal and construct validity. The heterogeneity of articles meant it was not possible to perform any meta-analysis.

**Conclusions:**

Previous studies have not well classified which facet of training or educational outcome the study is aiming to explore and had poor internal and construct validity. Future research should aim to validate a particular outcome measure, preferably by reproducing previous work rather than adopting new methods. In particular cognitive processing enhancement, demonstrated in a number of the medical student studies, should be tested at a postgraduate level.

## Background

There are a plethora of educational programmes and implementation strategies aimed at improving the quality of care delivered by health care professionals. A number of these are delivered via information technology systems with the use of video as an educational medium well established [1-3]. A new educational tool, that has become possible through multimedia advances in the last decade, is the audio-visual demonstration of signs and symptoms in patients, referred to as Patient Video Cases or PVCs [4]. They are easily displayed via online platforms, are widely used, and have been endorsed by the National Patient Safety Agency [5] as an example of good practice. However there is little academic study of their effectiveness. Given the financial pressures affecting all health care agencies, it is important to know if these resource intensive e-learning strategies give demonstrable benefit to patients or health care professionals.

Theoretical constructs exist to evaluate interventions designed to improve clinical performance, but no single approach is followed, due to the wide range of individual and organisational factors that affect the outcomes before, during and after the intervention [6]. Kirkpatrick’s training evaluation is defined by four distinct levels of outcome to be approached in a stepwise fashion [7]. The four key domains of the Kirkpatrick model are learner satisfaction, learner knowledge, learner behaviour change and organisational change. Although others have argued contextual factors not classified under these domains may be significant [6], the Kirkpatrick model still remains a valid methodology with systematic reviews using the process to examine training effectiveness [8]. A healthcare relevant modification of the Kirkpatrick model has been used in a study of inter-professional education in health and social care [9]. When using the Kirkpatrick model, or other relevant frameworks for assessing an educational or training intervention, the outcome measures and the methodology by which they are obtained must be valid. The concepts of internal and construct validity are classifications with direct relevance to outcome measures and are components of methodological quality used by the Campbell Collaboration [10,11].

i. Internal Validity *is the extent to which the intervention can reliably be ascribed to have affected the change*

ii. Construct Validity *relates to the association between the concept being investigated and the measures used to test it* i.e. does the data collected accurately reflect the outcome measure chosen?

Other forms of validity exist but are not directly relevant to the quality of the outcome measures chosen, for example good external validity would imply that using PVCs could be beneficial in different populations, but would not give any information if the initial outcome measure was fit for purpose.

The aim of this work is to answer the question “What is the validity and quality of outcome measures that have been used to evaluate interventions based on PVCs?”. This literature review will be used to identify which outcome measures are most valid in the assessment of the clinical effectiveness of an intervention based on PVCs. It will also help identify areas where more methodological research is needed to enable future studies to demonstrate high internal and construct validity.

## Methods

This review was performed over three stages, the first stage collating relevant literature followed by individual study quality appraisal in stage two with a summation of the overall validity of the studies.

### Stage one

Stage one identified literature relevant to the use of PVCs in health care settings. The definition of Health Care Settings used was; ‘any location or environment where students or graduates are practising or learning medicine.’ The definition of a PVC was; ‘any pre-recorded or live video footage of a patient used for the purposes of demonstrating a sign or symptom’. It did not include footage recorded for the purposes of educating other patients or families. Inclusion criteria were:

i. Humans

ii. The study described the use of PVCs in a training, educational (undergraduate or postgraduate), implementation capacity or environment.

As PVCs relate to demonstration of signs and symptoms in patients, studies using video to demonstrate verbal communication, non-lexical utterances or solely history taking between a patient and doctor or patient and patient were excluded as were non-English language papers which could not be translated. The full literature search was developed in conjunction with a senior NHS Librarian and is available on request. The following general search terms were used (Video* OR Video record* OR video clip OR digital* record* OR analogue recording OR *patient video clip)* and (Educat* OR Train* OR learn* OR teach* OR inservice training). The following databases were searched: Medline, British Nursing Index (BNI), EMBASE, Health Management Information Consortium (HMIC), CINAHL, NIHR Health Technology Assessment Programme (HTA), Database of Abstracts of Reviews of Effects (DARE), Scopus, The Cochrane Library and the Education Resources Information Centre (ERIC). Internet search engines and NHS evidence were used to identify publications or articles related to the search terms. The search strategy was not limited to any particular research methodology used in the articles. The last search performed was 27th^th^ July 2012 by the principal author. In all phases of the study any uncertainty as to classification or indexing of information was discussed with the collaborating authors.

Articles with a relevant abstract (any detail relating to the recording and utilisation of video clips of patients) had a complete paper review (as did any abstracts in which there was uncertainty about inclusion potential). Information on aim, health care user, educational purpose, modified Kirkpatrick training level domain, type of study, outcome measure and conclusions was extracted from each paper as shown in Table 1. The Educational purpose was subdivided into three categories:

**Table 1:** Studies by Healthcare professional grouping

**Health care professional**	**Number of studies pertaining to that group**
Undergraduates	11
Basic Postgraduate Training	3
Specialist Postgraduate Training	1
Undergraduate professionals allied to Medicine	2
Professional allied to Medicine	1
Trained Doctor Continuing Professional Development	2
Veterinary Students	1
**Total**	**21**

### Stage two

To enable objective review of articles to determine the aspects of validity under study the following domains were used which represent features reducing the internal validity of studies. They have been amended from the list described by Farrington [12]. This work was chosen as it is based on Cook and Cambell’s original work on methodological quality. Although other methodologies of analysis are available this is a widely used and accepted process which allows for an objective process to be applied.

1. Selection: Does the outcome measure allow for control between groups?

2. History: Does the outcome measure allow for the effects caused by some event occurring at the same time as the intervention?

3. Maturation: Does the outcome measure allow for natural progression in learning and knowledge?

4. Instrumentation: Is the outcome measure reproducible?

5. Testing: Does the outcome measure itself affect the results?

6. Differential attrition: Can the outcome measure control for differing numbers of participants in control or experimental groups (if present) or large drop out rates.

The extraction of information was undertaken by the principal author.

### Stage three

Once this process had occurred a number of more global questions were asked of each paper to determine whether the article’s author had evaluated the outcome methods they had chosen and allow an assessment of the construct validity of the study.

a) How was the choice of outcome measure justified?

b) Did the choice determine the results the study aims to investigate?

c) To what extent were the writers aware of the disadvantages as well as the advantages of the outcome measures chosen?

d) How did they overcome the disadvantages?

## Results

**Figure 1:**
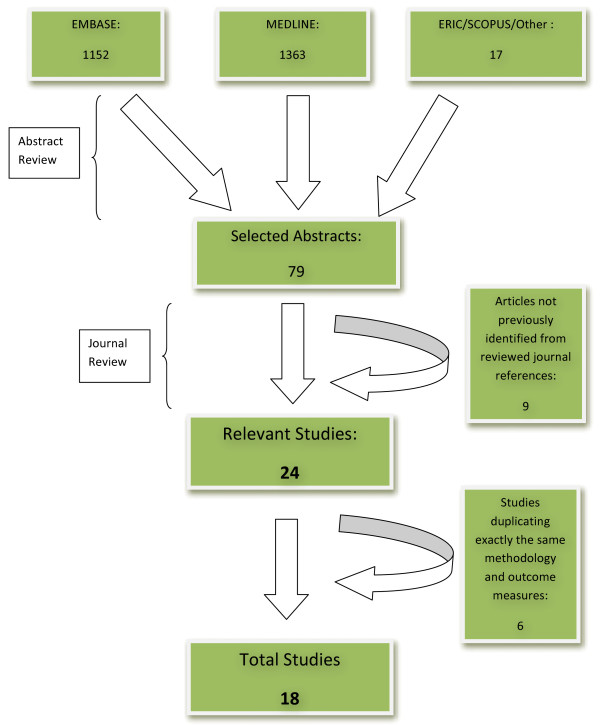
Literature Search Flow Diagram.

Figure 1 shows the flow of journals from the initial search to the final selection of articles. The types of healthcare professionals studied is demonstrated in Table 1 and the number of studies classified by educational purpose and Kirkpatrick level shown in Table 2. Two studies evaluated both undergraduate and basic postgraduate trainees leading to a total of 21 studies of health care professional groups and two studies evaluated both learner knowledge and learner behaviour leading to a total of 20 studies of the relevant Kirkpatrick level.

**Table 2:** Classification of studies

**Educational purpose**	**Number of studies**	**Kirkpatrick level**	**Number of studies**
Knowledge Gain	8	Learner Reaction (level 1)	5
Testing Methods	3	Learner Knowledge (2a)	0
Patient Examination Skills	5	Learner Knowledge (2b)	8
Overall Clinical Care	2	Learner Behaviour	7
**Total**	**18**	**Total**	**20**

The purpose of this work was to be as inclusive as possible so as to capture all outcome measures used. Although twenty-two articles (twenty-three studies) underwent a thorough analysis in stage two, half of these require further clarification as to the reasons for their inclusion. These articles were all reviewed by all three authors and a collaborative decision reached on their inclusion. Under the inclusion criteria it had not been the intention to include animal studies in the protocol. However one, in the field of veterinary medicine [13], studied PVCs in precisely the context human patients clips would be used with an accompanying relevant and feasible methodology. It has been included in the final review as it was decided methodology rather than context was being investigated. The search was repeated removing the ‘human only’ limitation but no other veterinary journals of relevance were found.

One study examining an intervention to improve the physical examination component of a medical student exam via a web-based video did not specifically use abnormal or normal clinical signs [14]. The study looked at outcomes across a whole year group in a *before and after cohort* design. This study has been included as the methodology could have been easily used in a PVC-related intervention. A study using video to demonstrate a specific clinical examination was also included although it could be argued that the precise aim of the tool was not demonstrating specific clinical signs but a methodology of elucidating them. The methodology used, a Solomon four-group design [15], was considered relevant to defining robust outcome measures in future PVC studies.

Finally six studies [16-21], although in different patient groups (ankylosing spondylitis, rheumatoid arthritis, fibromyalgia) used exactly the same methodology as two initial studies into osteoarthritis by the same investigators. These were studies in the validation of an examination methodology in both medical students and consultants. Although the actual data was different, the papers used exactly the same introduction, methods and discussion. In terms of the narrative review, these eight journal articles represent only one methodological approach in two different cohorts of participants. It was felt due to the lack of difference in the wording of the arthritic publications these should be considered as two studies, one representing undergraduates and the other trained doctors continuing professional development. Noting the reasons given above the total number of articles evaluated was 17 (which involved 18 distinct studies).

Table 3 contains the descriptive results for the reviewed articles and Table 4 contains the overall judgement on each of the articles. The analysis of the validity of the outcome measures can be found in the Additional file 1: Appendix.

**Table 3:** Identification of health care settings in which educational patient video clips have been utilised

**Paper**	**Aim or hypothesis**	**Health care user**	**Educational purpose**	**Kirkpatrick level**	**Type of study**	**Outcome methodology**	**Conclusions**
Using interactive video to add physical assessment data to computer based patient simulations [22]	Interactive video in patient simulations improves the learning experience [*Not formally Stated in paper*]	Basic Postgraduate Training	Overall Clinical Care	Learner Behaviour (level 3)	Comparative	Chart Review of interview and physical examination description of actual patients versus simulation performance	Good to fair agreement on overall comparison (kappa=0.72) and physical examinations (kappa=0.7)
The development of shared cognition in paediatric residents analysing a patient video versus a paper patient case [23]	Will supplementing a written case vignette by a PVC as opposed to an equivalent paper case increase shared cognition as measured by the frequency of collaborative concept link formation in the context of collaborative learning about movement disorders….?	Medical Students	Knowledge Gain	Leaner Knowledge (level 2b)	Cluster Randomised Control Trial	Identification and frequency of collaborative and individual concept links	The video group showed a significant increase (p<0.01) in collaborative concept links but not in individual concept links after watching the video
Enhancing diagnostic accuracy among non-experts through use of video cases [24]	(1) How does the level of diagnostic accuracy evolve through an interactive teamwork approach using PVCs?	Specialist Postgraduate Training	Knowledge Gain	Learner Knowledge (level 2b)	Repeated Measures Design	Analysis of frequency and of new diagnoses and new clinical reasoning processes as new information presented during review of PVC case.	i) New clinical reasoning processes were most frequent at first review of the PVC.
							ii) Frequency of new relevant diagnoses were stable at each step whereas less relevant diagnoses decreased.
	(2) Does the level of diagnostic accuracy differ between non-experts and experts?						
							iii) Relevant clinical reasoning was significantly higher amongst non-experts compared with experts at the small group discussion and think aloud procedure with content expert.
Introduction of patient video clips into computer-based testing: Effects on item statistics and reliability estimates [25]	To compare the basic characteristics and reliability of questions using video-based vignettes to questions using analogous text-based vignettes.	Medical Students	Testing Methods	Learner Knowledge (level 2b)	Parallel test questions with cross-over of video and text descriptors.	Median Item statistics and reliability estimates for test items	Overall, video-based questions had comparable difficulty and discrimination compared to analogous text-based questions.
Video-based test questions: A novel means of evaluation [26]	Video-Based Test items are supported by students [*Not formally stated by paper*]	Undergraduate professionals allied to Medicine	Testing Methods	Learner reaction (level 1)	(1) Questionnaire utilising repeated measures ANOVAs	Students preference between video-based and multiple choice questions	Students thought video based questions deepened understanding and recommended video-based questions be used in future exams.
Video-based test questions: A novel means of evaluation [26]	Unclear	Undergraduate professionals allied to Medicine	Testing Methods	Learner Knowledge (level 2b)	(2) Observational comparative study (One group informed about video questions the other not)	Exam scores in 12 video-based items	Students informed about video clips correctly answered more video based items
A comparison of critical thinking in groups of third-year medical students in text, video, and virtual PBL case modalities [27]	Critical Thinking, as exemplified by the discourse among students during group discussion, differs among groups receiving the same case with the same facilitator in one of three formats.	Medical Students	Knowledge Gain	Learner Knowledge (level 2b)	Three way comparative study (face-2-face with text, face-2-face with video, virtual with video)	Critical thinking discourse analysis	The virtual groups had the highest critical-thinking ratio. Except for the problem-identification stage, the video groups had higher ratios that the text groups did.
Comparison of text and video cases in a postgraduate problem-based learning format [28]	The addition of a video case to written information would lead to a greater increase in the frequency of data exploration, theory building and evaluation and metareasoning than would be a achieved by a paper case.	Medical Students	Knowledge Gain	Learner Knowledge (level 2b)	Randomised comparative study of video versus text cases	Frequency of pre-defined clause categories	The verbal interaction showed statistically significant improvements in data exploration, theory building and theory evaluation after the video case
Use of animation-enhanced video clips for teaching abnormal breathing patterns [13]	To gather feedback regarding the Animated Breathing Pattern Videotape	3^rd^ year **Veterinary** Students, House Officers and Faculty, Qualified Veterinarians	Patient Examination Skills	Leaner reaction (level 1)	Questionnaire	Usefulness and Satisfaction	Uniformly positive responses
Using web-based video to enhance physical examination skills in medical students [14]	To measure changes in first year students’ performance of physical examinations on standardized [sic] patients after implementation of a web-based curriculum	Medical Students	Patient Examination Skills	Learner Behaviour (level 3)	Before and After Cohort Outcome study	(i) Percent correct score in physical exam item checklist	Students on Web-based curriculum had higher level of competency and reduction in poor performance levels
						(ii)Mean score on physical exam process instrument	
Teaching the plantar reflex [15]	To test to efficacy of video-tape in the evaluation of the planter response	Medical Students	Patient Examination Skills	Learner Behaviour (level 3)	Solomon Four Group Design – Two experimental and control groups (with and without entrance test)	Correct judgement of graded presence of clinical sign	Small non-significant difference between experimental and control groups [evidence of sampling error]. If analysis was restricted to students who performed an entrance test there was a statistical significance in favour of the video group.
A videotape-based training method for improving the detection of depression in residents of long-term care facilities [29]	Does a training programme involving video based scenarios improve nursing staffs’ detection of depression within long-term care facilities? [Not formally stated by the paper]	Professionals allied to Medicine	Overall Clinical Care	Learner Knowledge (level 2b) and Learner Satisfaction (level 3)	Parallel group delayed intervention design.	(I) Videotape vignette test	Significant increase in performance in the intervention group which was maintained for at follow up for both vignette and written test.
						(ii) Written Test	
						(iii) Course evaluation questionnaire	
							Good levels of satisfaction on questionnaire
Advantages of video trigger in problem-base learning [30]	The reasons behind preferences for video triggers or paper cases in students and facilitators who are accustomed to paper cases.	Medical Students	Knowledge Gain	Learner Reaction (level 1)	Questionnaire	Usefulness and Satisfaction	Video triggers were preferred by both students and facilitators over paper cases in Problem Based Learning
A triangulated approach to the assessment of teaching in childhood epilepsy [31]	Evaluation of participant perceptions of learning	Medical Student s and Basic Postgraduate Training	Knowledge Gain	Learner Reaction (level 1)	Triangulation Outcome Analysis	Participant assessment (rating scales, open ended questions and focus groups), Lecturer reflection and peer observations	Videos identified as the most useful and interesting teaching tool. Results cross-validated by lecturer and peer observations
How video cases should be used as authentic stimuli in problem-based medical education [32]	To examine students views on the value of video cases compared to text based cases.	Medical Students	Knowledge Gain	Learner Reaction (level 1)	Focus Groups	Thematic Analysis	Video generally valuable but benefit dependant on certain conditions.
Visual expertise in paediatric neurology [33]	To investigate visual attention and cognitive processes of clinicians of varying degrees of experience diagnosing authentic paediatric video case	Medical Students, Basic Postgraduate Training and Consultant CPD	Knowledge Gain	Learner Knowledge (level 2b) and Behaviour (Level 3)	Observational study	Eye-tracking data were analysed with verbal recordings.	More experienced clinicians were more accurate in visual diagnosis and spent more of their time looking at relevant areas
An evaluation of the effectiveness of a videotape programme on inter-observer reliability in outcome assessment for osteoarthritis [34]	Whether interobserver variability in senior medical students could be reduced in a group of patients with OA using only a single viewing of an instructional videotape.	Medical Students	Patient Examinations Skills	Learner Behaviour (level 3)	Before and After Study [**Video intervention poorly described**]	Change in mean values of previously described observer dependant measures per participant	Pre-standardization reliability coefficients were <0.80 for seven measures. Coefficients for the performance of knee goniometry were uniformly low. Following the intervention, all but four reliability coefficients were >/= 0.93.
						Reliability coeffecients for the group	
An evaluation of the effectiveness of a videotape programme on inter-observer reliability in outcome assessment for fibromyalgia [16]	Whether interobserver variability in senior medical students could be reduced in a group of patients with fibromyalgia using only a single viewing of an instructional videotape.	Medical Students	Patient Examinations Skills	Learner Behaviour (level 3)	Before and After Study [**Video intervention poorly described**]	Change in mean values of previously described observer dependant measures per participant	Pre-standardization reliability coefficients were <0.80 for 8 measures. Following standardization all reliability coefficients, but one, approximated or exceeded 0.80
						Reliability coeffecients for the group	
An evaluation of the effectiveness of a videotape programme on inter-observer reliability in outcome assessment for ankylosing spondylitis [18]	Whether interobserver variability in senior medical students could be reduced in a group of patients with ankylosing spondylitis using only a single viewing of an instructional videotape.	Medical Students	Patient Examinations Skills	Learner Behaviour (level 3)	Before and After Study [**Video intervention poorly described**]	Change in mean values of previously described observer dependant measures per participant	Pre-standardization reliability coefficients were < 0.80 for three measures. Following standardization 12 reliability coefficients exceeded 0.80. For the majority of measures pre-standardization reliability coefficients were high and no further improvement in reliability could be demonstrated
						Reliability coeffecients for the group	
An evaluation of the effectiveness of a videotape programme on inter-observer reliability in outcome assessment for rheumatoid arthritis [17]	Whether interobserver variability in senior medical students could be reduced in a group of patients with rheumatoid arthritis using only a single viewing of an instructional videotape.	Medical Students	Patient Examinations Skills	Learner Behaviour (level 3)	Before and After Study [**Video intervention poorly described**]	Change in mean values of previously described observer dependant measures per participant	Pre-standardization reliability coefficients were >0.80 for all measures and remained above 0.80 following standardization except for one measure
						Reliability coeffecients for the group	
Osteoarthritis antirheumatic drug trials: Effects of a standardized instructional videotape on the reliability of observer-dependent dependent outcome measures [35]	Whether interobserver variability in consultants could be reduced in a group of patients with OA using only a single viewing of an instructional videotape.	Consultant CPD	Patient Examination Skills	Learner Behaviour (level 3)	Before and After Study [**Video intervention poorly described**]	Change in mean values of previously described observer dependant measures per participant	Prestandardization reliability coefficients were >0.80 for all measures and remained above 0.80 following the intervention
						Reliability coeffecients for the group	
Fibromyalgia antirheumatic drug trials: Effects of a standardized instructional videotape on the reliability of observer-dependent outcome measures [19]	Whether interobserver variability in consultants could be reduced in a group of patients with fibromyalgia using only a single viewing of an instructional videotape.	Consultant CPD	Patient Examinations Skills	Learner Behaviour (level 3)	Before and After Study [**Video intervention poorly described**]	Change in mean values of previously described observer dependant measures per participant	Prestandardization reliability coefficients were <0.80 for 8 measures. Following standardization all reliability coefficients approximated to or exceeded 0.80.
						Reliability coeffecients for the group	
Rheumatoid arthritis antirheumatic drug trials: Effects of a standardized instructional videotape on the reliability of observer-dependent outcome measures [20]	Whether interobserver variability in consultants could be reduced in a group of patients with rheumatoid arthritis using only a single viewing of an instructional videotape.	Consultant CPD	Patient Examinations Skills	Learner Behaviour (level 3)	Before and After Study [**Video intervention poorly described**]	Change in mean values of previously described observer dependant measures per participant	Prestandardization reliability coefficients were >0.80 for all measures and remained above 0.80 following standardization
						Reliability coeffecients for the group	
Ankylosing spondylitis antirheumatic drug trials: Effects of a standardized instructional viddeotape on the reliability of observer-dependent outcome measures [21]	Whether interobserver variability in consultants could be reduced in a group of patients with ankylosing spondylitis using only a single viewing of an instructional videotape.	Consultant CPD	Patient Examinations Skills	Learner Behaviour (level 3)	Before and After Study [**Video intervention poorly described**]	Change in mean values of previously described observer dependant measures per participant	Prestandardization reliability coefficients were <0.80 for three measures. Following standardization 12 reliability coefficients exceeded 0.80
						Reliability coeffecients for the group	

**Table 4:** Review of methodological quality of studies using outcome measures to assess the impact of PVCs

**Paper**	**1**	**2**	**3**	**4**		
	**How is the choice of outcome measure justified?**	**Will this choice determine the results the study aims to investigate?**	**To what extent are the writers aware of the disadvantages as well as the advantages of the outcome measures chosen?**	**How do they overcome the disadvantages?**	**Internal validity?**	**Construct validity?**
Using interactive video to add physical assessment data to computer based patient simulations [22]	Used to justify criterion validity of the intervention used.	Study aims not clear. If presumed to be to elucidate whether the simulations are an effective learning experience the outcome used partially confirms the simulation represent normal practice not that the simulation improved performance or was an useful education tool	Author notes that in actual practice chart noting is done under time pressure whereas with this simulation there was more time available to make case-notes more complete.	No comment made on this. The absence of details on whether a specific proforma for extracting information from the case-notes was used makes it difficult to assess how comparisons were made.	No	No
The development of shared cognition in paediatric residents analysing a patient video versus a paper patient case [23]	Process of capturing concept link formation described with the reason for using verbal protocol analysis supported by published evidence.	Yes	Authors note a small number of participants and only one case so reliability may be questioned.	The positioning of the simulated recall exercise straight after the group work limits loss of content due to degradation of memories.	Yes	Yes
			They also note the ability to accurately recall and record all concept links is not established.			
	Methodology of using simulated recall in individuals following the group discussions not well supported.					
				Authors comment although not blinded the interviewers were not recording cognitive processes just the thoughts that lead to them.		
			They were aware the interviewers were not blinded to the intervention group of the participants.			
Enhancing diagnostic accuracy among non-experts through use of video cases [24]	Previous work by the author has shown improved cognitive processes when PVCs utilised. Improved diagnostic accuracy is the natural conclusion of relevant or improved cognitive processing.	Yes	Study acknowledges the diagnostic accuracy as an outcome is only a short term variable of learning.	Authors argue increase in diagnostic reasoning in non-experts in may promote further literature study and learning.	Yes	Yes
			Authors touch on, but don’t specifically note, the outcome measure is not directly related to the intervention rather the group discussion following the intervention.			
Introduction of patient video clips into computer-based testing: Effects on item statistics and reliability estimates [25]	No justification for answer analysis is given although standard methodology applied.	Yes	Problems with questions with low discrimination values identified and subject to supplemental analysis.	Items with RPB values of zero or less than 0.2 removed although no explanation of why these values were chosen.	Not Applicable	Not Applicable
Video-based test questions: A novel means of evaluation [26]	No justification for questionnaire methodology given although this format is an accepted primary approach to gathering information on satisfaction with a process.	Yes although reliability of results must be treated with caution.	No comments made	No comments made	Not Applicable	Not Applicable
Video-based test questions: A novel means of evaluation [26]	No justification given	Aims of the study not clear	No comments made	No comments made	Not Applicable	Not Applicable
A comparison of critical thinking in groups of third-year medical students in text, video, and virtual PBL case modalities [27]	There is a theoretical association between Problem Based Learning and critical thinking. A sound research framework exists to analyse discourse and code for content. Therefore a process, discourse analysis , exists to examine the outcomes of PBL in respect to critical thinking.	Yes	The outcome measure depends on the validity and reliability of the coder.	Only one author did all the coding but coding agreement was checked using a sample of transcripts with two others (one not involved in the study).	Yes	Yes
			Although the coder is blind to group type it is possible for this to be suggested by the dialogue.			
			Time pressures on face-to-face groups may limit opportunities to refine critical thinking compared to the virtual groups.			
Comparison of text and video cases in a postgraduate problem-based learning format [28]	A coding system for cognitive and metacognitive thinking has been established. It is theoretically plausible a video case would improve thinking processes.	Yes. Although the actual reason for improved educational outcome in PBL has yet to be defined and the coding schema chosen is only one way of evaluating cognitive and metacognitive processes.	The outcome measure itself is not examined although the article notes the use of one author for both groups and the low numbers of residents in both groups mean caution is required in interpretation.	The use of clause frequency enables variability in group sizes to be addressed.	Yes	Yes
Use of animation-enhanced video clips for teaching abnormal breathing patterns [13]	No justification given for questionnaire methodology although this format is an accepted primary approach to gathering information on satisfaction with a process.	Yes although the reliability of results must be treated with caution.	No comment made. The authors note that some respondents gave inconsistent written comments when compared with their agreement with statements and comment it was likely they had mis-understood the question.	No comments made	Yes	No
Using web-based video to enhance physical examination skills in medical students [14]	Summative clinical skills assessment has been utilised (and presumably validated although this is not stated) in the learning institution the study is taking place in.	Yes, although dependant on the reliability of the Clinical Skills Assessment.	The authors comment they did not track the utilisation of the video clip website by students and note a prospective, randomised controlled study would have been more accurate.	No comments made	Potentially Not	Yes
Teaching the plantar reflex [15]	No justification given. Process for assessing performance described although the standardised rating scale was not demonstrated.	Yes but only if the assessment system is valid.	The authors acknowledge the effect of the entrance test in providing education in itself.	No comment made	Potentially Not	Yes
A videotape-based training method for improving the detection of depression in residents of long-term care facilities [29]	Outcome measures well described but no comment on the reason for using them.	As patient outcome not measured methodology can only assess how the training programme improves performance in the outcomes tested.	Authors not a large sample size is needed to counter affects of attrition.	Authors tested after a control period and withheld feedback to participants about their test performances.	Potentially Not	Yes
			They also not the vignette video test may improve practice in its own right.			
			Staff were allowed to choose timing and type of session according to their needs with no control for group or individual sessions.			
Advantages of video trigger in problem-base learning [30]	No justification for questionnaire methodology given although this format is an accepted primary approach to gathering information on satisfaction with a process.	Yes as long as sample of participants valid.	No comments made	No comments made	Yes	Yes
A triangulated approach to the assessment of teaching in childhood epilepsy [31]	Triangulation used as a more complete and robust measure to validate findings.	Yes although must be employed in a methodological fashion. However the use of video clips was not the sole purpose of the study and questions not posed to determine this.	Very little attention paid to confounding influences and the fact that the cross –validation was not particular well demonstrated.	No comments made	No	Not applicable
How video cases should be used as authentic stimuli in problem-based medical education [32]	Focus groups a well refined qualitative tool which all deep analysis of concepts presented.	Yes	Clear acknowledgement of the problems with individuals dominating or evading group discussion.	Very experienced facilitator used	Yes	Yes
Visual expertise in paediatric neurology [33]	Important differences in perception between experts and novices studying dynamic stimuli has been documented . Authors note this field is underexplored in the medical domain but use a high quality eye tracking machine and linked to spoken cognitive processes	Yes	Note that outcome method was novel and made efforts to triangulate findings to gold standard outcomes (such as correct diagnosis)	Used variety of experience in subject population	Yes	Yes
An evaluation of the effectiveness of a videotape programme on inter-observer reliability in outcome assessment for osteoarthritis [34]	Outcome measure used in previous studies to assess performance in musculo-skeletal examination.	Yes	A larger matrix and more observers and patients may have been used to improve reliability.	The authors own previous work has indicated the 6x6 is pragmatic and representative.	No	Yes
Osteoarthritis antirheumatic drug trials: Effects of a standardized instructional videotape on the reliability of observer-dependent dependent outcome measures [35]	Outcome measure used in previous studies to assess performance in musculo-skeletal examination.	Yes	A larger matrix and more observers and patients may have been used to improve reliability.	The authors own previous work has indicated the 6x6 is pragmatic and representative.	No	Yes

## Discussion

This review examined the evidence on how to measure outcomes when Patient Video Cases (PVCs) are used in healthcare settings. This evidence was small, extremely heterogeneous and there was insufficient evidence to specify the best outcomes to use. The heterogeneity in the articles was created by the diversity of involved health care professionals, varying educational purposes, different types of intervention, a wide range of outcome methodologies, different internal and construct validities and a variety of results. Each of these is examined in turn.

### Type of healthcare professional

The preponderance of projects in undergraduate education is likely related to the large number of medical education academics at these institutions, the access to a ‘captive group’ of subjects and the greater ease of assessing undergraduate outcomes. Further investigation into the use of PVCs at postgraduate level and in other healthcare professionals is clearly warranted. For all health care professionals it is also reasonable to attribute the lack of studies to the difficulties in designing [36] and funding studies evaluating PVCs.

### Educational purposes and types of intervention

Given the small number of studies, it is difficult to identify clear treads in educational purpose or type of intervention. Learner satisfaction and knowledge gain are the easiest of the Kirkpatrick training outcomes to measure as they do not require external observation or intervention. However these domains are the lowest in the hierarchy of evidence needed to confirm that a training process has been truly effective [37]. No study looked at organisational change, which is in keeping with previous literature. A review aiming to identify methods used to measure change in the clinical practices of health professionals found only 17.6% looked at changes at an organisation level [38]. Also in this review only one study attempted to look at more than one level of training outcome. A systematic review of evaluation in formal continuing medical education [39] noted 28% of studies reviewed looked at two levels and only 6% looked at three.

### Methods for determining and assessing outcome measures

Reflecting the wide range of different types of studies performed, the validity of the outcome measures used was variable. This represents the difficulties of examining interventions related to education and training. In clinical practice a gold standard approach in assessing the effectiveness of medication is the randomised controlled trial. The primary outcome measure being an objective endpoint such as a defined reduction or gain in a physiological parameter. In training interventions, a single endpoint as an outcome requires a lot of interpretation, and potential criticism. For example, learner satisfaction does not necessarily equate to knowledge change, neither does it have a direct correlation with change in practice. The absence of a gold standard measure to assess training interventions may have led researchers to be opportunistic in their use of outcome measures. In this review seven studies gave no justification for the outcome measure used [13,15,25,26,29,30]. In addition comments by the authors themselves on limitations to the outcome measures were absent in five of the studies [13,26,30,31].

Only one study looked at more than one discrete domain in the Kirkpatrick training evaluation framework [29]. In this work both learner knowledge and learner satisfaction were assessed by different measures (a video test, a written test and a course evaluation). Three other studies [14,25,31] had more than one outcome measure, although these were all subtle variations on a theme such as scores in different types of clinical examination in the same test.

Only two of the studies [27,33] satisfied all domains when deciding on whether internal and construct validity had been achieved. Three other papers [15,23,29] had minor concerns, generally relating to the extent which the outcome measure itself affected the results. Questionnaire studies reflecting learner satisfaction tended not to perform well as control between groups was not possible and confounding factors were very difficult to assess.

### Results of the interventions

Nearly all papers were positive regarding the use of PVCs (regardless of whether the analysis above had revealed concerns over the validity of the outcome measure). The medical student studies regarding critical analysis and thinking showed strong results in favour of the use of PVCs. The underlying hypotheses of these studies [23,24,27,28,32] were plausible and the methodologies used rigorous. A researcher independent of these groups has also recently shown students prefer this use of PVCs to current problem based learning techniques [30] so triangulation has in some respects been achieved in this field. A recent paper demonstrating experts are more focused on the relevant clinical features within patient video clips has been further supported by, as yet unpublished evidence, that eye movement modelling may improve diagnostic reasoning. This methodology, where the minute movements of the eye are tracked while observing dynamic images, has strong construct validity. It is felt the cognitive ‘load’ of dynamic video clips may encourage cognitive processing [40] and therefore methodologies to explore the extent of this load created by PVCs are welcome. Future research must be cognisant of the fact that under- or over-load may occur depending on the capacity of the individual engaging in the activity. Extraneous cognitive [41] load may be able to be controlled to some extent by investigators and this will aid determination of its impact on the outcome of the intervention.

Studies concerning testing methods and clinical examination showed no obvious differences between PVCs and current assessment methods. The potential difficulty and cost of placing video clips into examinations (whether formative or summative) may have limited the number of validation studies in this area. In studies of clinical examination technique which aimed to show improvement following a PVC intervention, there was supportive evidence although initial skill sets tended to be relatively high. The importance of controlling for this was demonstrated by the use of the Solomon Four Group design on a video intervention to improve examination of the plantar reflex [15]. In this study an effect was only seen when pre-intervention performance was assessed.

The video-based training method for improving the detection of depression in residents of long term care facilities demonstrated an increase in performance of the intervention group in both knowledge assessments [29]. Direct patient benefit was not assessed so an improvement in clinical care as a use of PVC cannot be claimed. However given the good levels of satisfaction on questionnaire testing it is likely that participants would not have been averse to incorporating newly acquired learning into their day-to-day practice.

### Limitations

The heterogeneity of the current published evidence made a robust narrative review extremely difficult. Apart from the work on how PVCs encourage discourse and critical thinking, there were no common themes in which to be able to extract information and analyse composite outcomes. This may represent difficulty in undertaking research in the field (the cost of production of video clips), the difficulty in defining valid outcome measures or publication bias due to a paucity of positive outcomes. This exemplifies the challenge that much medical education research is Action Research, research based on the instructors’ own practice.

Publication bias is unlikely to be significant as there as there is literature in which research is positive [42] regarding the use of video and online technologies but there are also negative [43] publications in existence. It would seem unlikely a particular modality of online or audiovisual learning would be subject to a different research agenda.

The main limitation of this study is the low number of articles that were found. The search strategy used was expansive although “Patient Video Clip” or similar terms are not used by all researchers in the field. It is possible terms other than those searched have been used although the number of papers missed is likely to be very small. Extraction of data was performed by a sole reviewer so it is possible so errors of typology were made although the small number of final articles has allowed extensive examination of the papers by all the authors.

## Conclusion

This review process has demonstrated the diverse nature of research in determining the effectiveness of PVCs in education. Medical education occurs in a variety of environments and the complicated interplay of confounding variables makes interpretation of outcomes difficult. The following recommendations would enable the production of a standard conceptual framework to guide future research in the area. 

• Studies should classify which facet of training or educational outcome the study is aiming to explore.

• Studies should aim to validate a particular outcome measure, preferably by reproducing previous work rather than adopting new methods.

• A description of the validity of the chosen outcome measure should be included in study protocol.

• Although control groups are useful for demonstrating the benefit of a PVC intervention, more evidence is needed on whether the outcome measure demonstrates construct validity.

• Studies on PVCs should take account of cognitive theory with the cognitive processing enhancement, demonstrated in a number of the medical student papers, tested at a postgraduate level. Although pragmatic outcome measures are easier to achieve explanatory trials are needed.

• Prior-knowledge and behaviour testing is vital to demonstrate improvement.

## Competing interests

The author(s) declare that they have no competing interests.

This report is independent research arising from a Doctoral Research Fellowship supported by the National Institute for Health Research. The views expressed in this publication are those of the author(s) and not necessarily those of the NHS, the National Institute for Health Research or the Department of Health.

## Authors' contributions

DR proposed the original research concept, carried out the literature review and prepared a first draft. DM and TC made significant contributions to the design and methodology of the review as well as to subsequent drafting of the article. All authors read and approved the final manuscript.

## Pre-publication history

The pre-publication history for this paper can be accessed here:

http://www.biomedcentral.com/1472-6920/12/125/prepub

## Supplementary Material

Additional file 1 **Appendix.** Review of studies to assist in evaluation of internal and construct validity based on framework via Farringdon. Click here for file
