# Supplementary material for: Towards a conceptual framework demonstrating the effectiveness of audiovisual patient descriptions (patient video cases): a review of the current literature
Source: BMC Med Educ. 2012 Dec 21;12:125. doi: 10.1186/1472-6920-12-125 (PMC3542158; doi:10.1186/1472-6920-12-125)
Supplement: Additional file 1 — Appendix. Review of studies to assist in evaluation of internal and construct validity based on framework via Farringdon. [file 1472-6920-12-125-S1.docx]

**Appendix –Review of studies to assist in evaluation of internal and construct validity based on framework via Farringdon**

**[Due to repetition of content articles 16,17,18,20,21,22 from table four have been removed]**

| **Paper** | **Selection** | **History** | **Maturation** | **Instrumentation** | **Testing** | **Differential Attrition** |
| --- | --- | --- | --- | --- | --- | --- |
|  | **Does the outcome measure allow for control between groups?** | **Does the outcome measure allow for the effects caused by other events?** | **Does the outcome measure allow for natural progression of learning?** | **Is the outcome measure reproducible?** | **Does the outcome measure itself affect the results?** | **Can the outcome measure control for differing numbers of participants in control or experimental groups (or drop outs)?** |
| Using interactive video to add physical assessment data to computer based patient simulations[1] | No. Case notes reviewed of individuals consulting simulated patients and actual patients but data extraction methodology not described. | No. Authors commented that chart notes on actual patients were much shorter than the more complete notes made on simulated patients. | Not applicable | Not without a defined case-note extraction methodology | Study did not comment on whether the participants were aware their notes were going to be analysed. | With a defined extraction methodology this would be possible. |
| The development of shared cognition in paediatric residents analysing a patient video versus a paper patient case[2] | Yes. A standardised procedure is described. | Yes. The stimulated recall exercise will enable other causes to be identified. | Yes. although in this study the outcome measure was examined during the learning exercise. Longer term evaluation was not performed. | Yes. Video of group discussions allows for third party validation of the concept links extracted and this is further refined by the stimulated recall exercise. | Potentially. The interaction of interviewer with the resident during the stimulated recall exercise may promote learning above which was occurring during the vignette review. | If saturation of themes was achieved then evaluation of differing numbers of participants would still be valid. |
| Enhancing diagnostic accuracy among non-experts through use of video cases[3] | No controls used in the study but the methodology could be used in control-group setting. | Yes as long as the intervention is controlled for. Clinical reasoning is explored but difficult to attribute directly to the intervention. | Yes although in this study the outcome measure was examined during the learning exercise. Longer term evaluation was not performed. | Yes. The recording and evaluation of new diagnoses and clinical reasoning processes can be performed objectively. | Potentially. The act of recording diagnoses and clinical reasoning may refine cognitive process during subsequent repeated measures. | Yes. Confidence intervals on the basis of a normal approximation of the poisson distribution were described. |
| Introduction of patient video clips into computer-based testing: Effects on item statistics and reliability estimates[4] | Yes. Discrimination Statistics performed in same manner in both groups. | No. Outcome measure used to determine whether participants knowledge dependant on format of question not the reasons underpinning the knowledge. | Not applicable | Yes. Standardised discrimination analysis. | No. | Statistical tests utilised should control for response rates |
| Video-based test questions: A novel means of evaluation[5] | Questions asked in both intervention and control groups. | No. Responses to questions may be affected by learning styles. | Potentially. Questions focused on the utility of the video clips rather than the knowledge contained within them | Yes. | No. | There may need to be minimum numbers of respondents in both groups. |
| Video-based test questions: A novel means of evaluation[5] | Insufficient detail on the production of the exam questions | No. Tests scores affected by many factors. | No. | Potentially but methodology of question production not described. | No. | There may need to be minimum numbers of respondents in both groups. |
| A comparison of critical thinking in groups of third-year medical students in text, video, and virtual PBL case modalities[6] | Yes. Objective scoring system for coding critical thinking stages although coding agreement may be variable depending on experience of coder. | Yes. Critical Thinking codes include analysis of source of learning or knowledge. | Yes. Critical Thinking codes include analysis of source of learning or knowledge. | Yes. although agreement in coding must be robust. | No. | There may need to be minimum numbers of respondents in both groups. |
| Comparison of text and video cases in a postgraduate problem-based learning format[7] | In this particular study one of the authors tutored both groups but this could be avoided in future studies. | No although content analysis may assist with this. | Uncertain. Increase in clauses related to improved cognitive processes was not tested over time. | Yes. although agreement in coding system for clause analysis must be robust. | No. | Clause Frequency Ratio allows for differences in group sizes. Low numbers of participants may not be representative however. |
| Use of animation-enhanced video clips for teaching abnormal breathing patterns[8] | No. Questions specific to audiovisual methodologies | No. Questions specific to the effect of the video clips | Potentially. Questions focused on the utility of the video clips rather than the knowledge contained within them | Yes. | No. | There may need to be minimum numbers of respondents in both groups. |
| Using web-based video to enhance physical examination skills in medical students[9] | Yes. Both physical Exam checklist and. General Assessment Scale can be applied to groups | No. Results may be dependent on many factors. | No. | Physical Exam Checklist is objective however the General Assessment Scale would require validation and control between assessors. | If the scoring checklist is available prior to the exam then the participants may use the checklist rather than the intervention to improve performance. | There may need to be minimum numbers of respondents in both groups. |
| Teaching the plantar reflex[10] | Yes. Solomon Four Group Design Used. | No. However the Solomon Group Design does enable base line differences in knowledge to be examined. | Yes. The Solomon Group Design enables base line differences in knowledge to be examined. | Yes although gold standard outcomes needs to be as objective as possible (in this study judgement against senior neurologist used) | Yes. Those with poor levels of knowledge are likely to improve at a greater rate when the correct approach is demonstrated at the entrance test. | There may need to be minimum numbers of respondents in both groups. |
| A videotape-based training method for improving the detection of depression in residents of long-term care facilities[11] | Yes. | Partially. Two outcomes assessed and delayed intervention approach used at control site. | Yes. Delayed intervention approach used. | Yes although video vignettes must be utilised equitably between groups. | Potentially. Video vignettes and Knowledge tests may encourage active learning by participants in the delayed intervention group. | There may need to be minimum numbers of respondents in both groups. |
| Advantages of video trigger in problem-base learning[12] | No. Questions specific to video triggers. | No. | No | Yes | No | There may need to be minimum numbers of respondents in both groups. |
| A triangulated approach to the assessment of teaching in childhood epilepsy[13] | No. Questions specific to interventions used in the lecture. | No. Unclear as to how long after intervention the questionnaire and focus groups were performed. Not clear if focus group aimed to determine if learning was possible from other sources. | No | Yes | No. Although reflection by the lecturer during the course of the study is likely to influence teaching style. | Yes |
| How video cases should be used as authentic stimuli in problem-based medical education[14] | No. Questions specific to intervention used. | Yes. | Yes. Focus groups can explore reasons for knowledge gain. | No. Partially dependant on facilitator. | Focus groups may prompt reflection which may alter responses. | No (unless good group sizes initially) |
| Visual expertise in paediatric neurology[15] | Yes | Yes (but external events not relevant with this methodology) | Yes as recording of spoken cognitive process would give clues to this. | Yes | No | There may need to be minimum numbers of respondents in both groups. |
| An evaluation of the effectiveness of a videotape programme on inter-observer reliability in outcome assessment for osteoarthritis[16] | Yes although in this case a control group was not used. | No although the time period between the intervention and the repeat testing was very short. | No. It is possible the before testing improved performance in the subsequent post intervention test. | Yes. The Standards are based on internationally agreed examination guidelines. A Latin Square design was used to reduce variability in position on the examination circuit. | Yes. The pre-test experience may influence the post-test results | There may need to be minimum numbers of respondents (and patients to examined) in both groups. |
| Osteoarthritis antirheumatic drug trials: Effects of a standardized instructional videotape on the reliability of observer-dependent dependent outcome measures[17] | Yes although in this case a control group was not used. | No although the time period between the intervention and the repeat testing was very short. | No. It is possible the before testing improved performance in the subsequent post intervention test. | Yes. The Standards are based on internationally agreed examination guidelines. A Latin Square design was used to reduce variability in position on the examination circuit. | Yes. The pre-test experience may influence the post-test results | There may need to be minimum numbers of respondents (and patients to examined) in both groups. |

References

1. White JE: **Using interactive video to add physical assessment data to computer-based patient simulations in nursing.** Comput Nurs 1995, **13**(5):233-235.

2. Balslev T, de Grave W, Muijtjens AMM, Eika B, Scherpbier AJJA: **The development of shared cognition in paediatric residents analysing a patient video versus a paper patient case.** Advances in Health Sciences Education 2009, **14**(4):557-565.

3. Balslev T, De Grave WS, Muijtjens AMM, Scherpbier AJJA: **Enhancing diagnostic accuracy among nonexperts through use of video cases.** Pediatrics 2010, **125**(3).

4. Lieberman SA, Frye AW, Litwins SD, Rasmusson KA, Boulet JR: **Introduction of patient video clips into computer-based testing: Effects on item statistics and reliability estimates.** Academic Medicine 2003, **78**(10 SUPPL.).

5. Hertenstein MJ, Wayand JF: **Video-based test questions: A novel means of evaluation.** Journal of Instructional Psychology 2008, **35**(2):188-191.

6. Kamin C, O'Sullivan P, Deterding R, Younger M: **A comparison of critical thinking in groups of third-year medical students in text, video, and virtual PBL case modalities.** Acad Med 2003, **78**(2):204-211.

7. Balslev T, de Grave WS, Muijtjens AM, Scherpbier AJ: **Comparison of text and video cases in a postgraduate problem-based learning format.** Med Educ 2005, **39**(11):1086-92.

8. Hawkins EC, Hansen B, Bunch BL: **Use of animation-enhanced video clips for teaching abnormal breathing patterns.** Journal of Veterinary Medical Education 2003, **30**(1):73-7.

9. Orientale E,Jr, Kosowicz L, Alerte A, Pfeiffer C, Harrington K, Palley J, Brown S, Sapieha-Yanchak T: **Using web-based video to enhance physical examination skills in medical students.** Fam Med 2008, **40**(7):471-6.

10. Raijmakers PG, Cabezas MC, Smal JA, van Gijn J: **Teaching the plantar reflex.** Clinical Neurology & Neurosurgery 1991, **93**(3):201-4.

11. Wood S, Cummings JL, Schnelle B, Stephens M: **A videotape-based training method for improving the detection of depression in residents of long-term care facilities.** Gerontologist 2002, **42**(1):114-21.

12. Chan LK, Patil NG, Chen JY, Lam JC, Lau CS, Ip MS: **Advantages of video trigger in problem-based learning.** Med Teach 2010, **32**(9):760-5.

13. Bye AM, Connolly AM, Netherton C, Looker P, Burgess A, Lonergan A: **A triangulated approach to the assessment of teaching in childhood epilepsy.** Med Teach 2007, **29**(2-3):255-257.

14. de Leng B, Dolmans D, van de Wiel M, Muijtjens A, van der Vleuten C: **How video cases should be used as authentic stimuli in problem-based medical education.** Med Educ 2007, **41**(2):181-8.

15. Balslev T, Jarodzka H, Holmqvist K, de W, Muijtjens AM, Eika B, van J, Scherpbier AJ: **Visual expertise in paediatric neurology.** European Journal of Paediatric Neurology 2012, **16**(2):161-6.

16. Bellamy N., Anjema C., Alikhan N., Chhina T., Dhanoa D., Edelist D., Esufali Z., Ismail F., Hill J., Campbell J.: **An evaluation of the effectiveness of a videotape programme on interobserver reliability in outcome assessment for osteoarthritis.** Inflammopharmacology 1999, **7**(2):143-154.

17. Bellamy N, Bachmeier C, Brooks P, Browne C, Cohen M, March L, Conaghan P, Day R, Campbell J: **Osteoarthritis antirheumatic drug trials: Effects of a standardized instructional videotape on the reliability of observer-dependent dependent outcome measures.** *Inflammopharmacology* **5:**.
